# Cellular Metrology: Scoping for a Value Proposition in Extra- and Intracellular Measurements

**DOI:** 10.3389/fbioe.2019.00456

**Published:** 2020-01-14

**Authors:** Nilofar Faruqui, Andreas Kummrow, Boqiang Fu, Carla Divieto, Fabiola Rojas, Florence Kisulu, Janaina J. V. Cavalcante, Jing Wang, Jonathan Campbell, Juliana L. Martins, Jun-Hyuk Choi, Maria Paola Sassi, Massimo Zucco, Maxim Vonsky, Sandrine Vessillier, Shan Zou, Shin-Ichiro Fujii, Maxim G. Ryadnov

**Affiliations:** ^1^National Physical Laboratory, Teddington, United Kingdom; ^2^Physikalisch Technische Bundesanstalt, Berlin, Germany; ^3^National Institute of Metrology, Beijing, China; ^4^Istituto Nazionale di Ricerca Metrologica, Turin, Italy; ^5^Instituto de Salud Pública de Chile, Santiago, Chile; ^6^Kenya Bureau of Standards, Mombasa, Kenya; ^7^National Institute of Metrology, Quality and Technology (INMETRO), Rio de Janeiro, Brazil; ^8^National Measurement Laboratory at LGC, Teddington, United Kingdom; ^9^Korea Research Institute of Standards and Science, Daejeon, South Korea; ^10^D. I. Mendeleyev Institute for Metrology, Saint Petersburg, Russia; ^11^Institute of Cytology, Russian Academy of Sciences, Saint Petersburg, Russia; ^12^National Institute for Biological Standards and Control, Potters Bar, United Kingdom; ^13^Metrology Research Centre, National Research Council, Ottawa, ON, Canada; ^14^National Metrology Institute of Japan, Tsukuba, Japan

**Keywords:** traceability, standards, cell metrology, regenerative medecine, stem cells, gene therapies

## Abstract

The symptomatic irreproducibility of data in biomedicine and biotechnology prompts the need for higher order measurements of cells in their native and near-native environments. Such measurements may support the adoption of new technologies as well as the development of research programs across different sectors including healthcare and clinic, environmental control and national security. With an increasing demand for reliable cell-based products and services, cellular metrology is poised to help address current and emerging measurement challenges faced by end-users. However, metrological foundations in cell analysis remain sparse and significant advances are necessary to keep pace with the needs of modern medicine and industry. Herein we discuss a role of metrology in cell and cell-related R&D activities to underpin growing international measurement capabilities. Relevant measurands are outlined and the lack of reference methods and materials, particularly those based on functional cell responses in native environments, is highlighted. The status quo and current challenges in cellular measurements are discussed in the light of metrological traceability in cell analysis and applications (e.g., a functional cell count). An emphasis is made on the consistency of measurement results independent of the analytical platform used, high confidence in data quality vs. quantity, scale of measurements and issues of building infrastructure for end-users.

## Background

Researchers working in the life sciences sector strive to provide solutions to societal challenges ranging from tissue restoration and generic disorders to cancer and microbial diagnostics. The sector is burgeoning, while the recent progress acknowledges the lack of standards that are necessary to guide diverse stakeholders and foster an environment for adherence (Freedman, 2013). The National Measurement Institutes (NMIs) invest a concerted effort to create mesurement assurance methods and underpin measurement systems with traceabile standards. This is critical to helping to translate an extensive body of research knowledge into commercial products in this and other industry sectors. Improving reproducibility and traceability in measurement results will conform to the competence requirements of bio-measurement laboratories and assure confidence in research (Thelen et al., 2019). However, “irreproducibility” is symptomatic of far broader challenges in biological measurements that cannot be addressed by an individual method, technique or material (Plant et al., 2014). Reference materials, methods, protocols and appropriate documentary standards are necessary (Freedman and Inglese, 2014). Each of these can address a specific measurand, but to describe a cellular process completely measurements must be validated in a continuum. Cross validation between different measurands can mitigate the problem of confounding variables in “noisy” cellular environments, which contribute to the notorious complexity of cellular systems (Freedman, 2015). Currently, SI units do not cover it fully: based on existing capabilities and regulatory requirements, many measurements are made in arbitrary units that do not necessarily allow for comparison across studies. In addition, biological measurements are often performed to determine nominal properties such as color obtained by Gram staining. However, even if one focuses on measurements that could in principle be SI traceable, gaps remain in the continuum of characterized cellular properties across length and time scales. Filling these gaps, while revealing different sources of uncertainty, is expected to support the provision of a complete cell metrology framework that can ultimately increase confidence in research. Furthermore, cell metrology can demonstrate value in emerging areas of global importance including regenerative medicine, infectious disease, eukaryotic and prokaryotic cell therapeutics as well as gene therapies to accelerate the translation of advanced medicinal products.

## Metrology Beginnings In Cell Analysis

Cells are hierarchal living systems and pose measurement challenges that are distinct from those of chemical and physical metrology. Metrology concepts and expertise, which are of second nature to chemistry and physics, are starting to gain momentum in biology. In part, this owes to the recent technical advancements that allow more precise and accurate measurements at the single-cell and population levels (e.g., atomic force microscopy, microfluidics and time-lapse microscopy, high-resolution flow cytometry). In part, this is because metrology developments in life sciences have an increasing impact on emerging technologies (Freedman, 2013). Cell analysis including intra-, inter-, sub- and extra-cellular measurements prompts the need to project where and what measurement science is necessary to assure comparability (Lin-Gibson et al., 2016b). Recognizing the importance of this, the Consultative Committee on the Quantity of Substance (CCQM) has recently established a Cell Analysis Working Group (CAWG) whose mission is to identify, establish and underpin global comparability of cell measurement capabilities through reference measurement systems of the highest possible metrological order with traceability to the SI or to other internationally agreed units. The group benchmarks claimed competences of the National Measurement Institutes for measurement services in the quantification of intact cells and cell properties. This strategy is driven toward transforming trial-and-error approaches used in translational research into more predictive solutions. The mission however faces two main challenges:

- incompleteness and inconsistency in the metrology of relationships between cellular structure, behavior and function, and
- a lack of defined measurands, reference methods and materials necessary for establishing such relationships.

**Figure 1:**
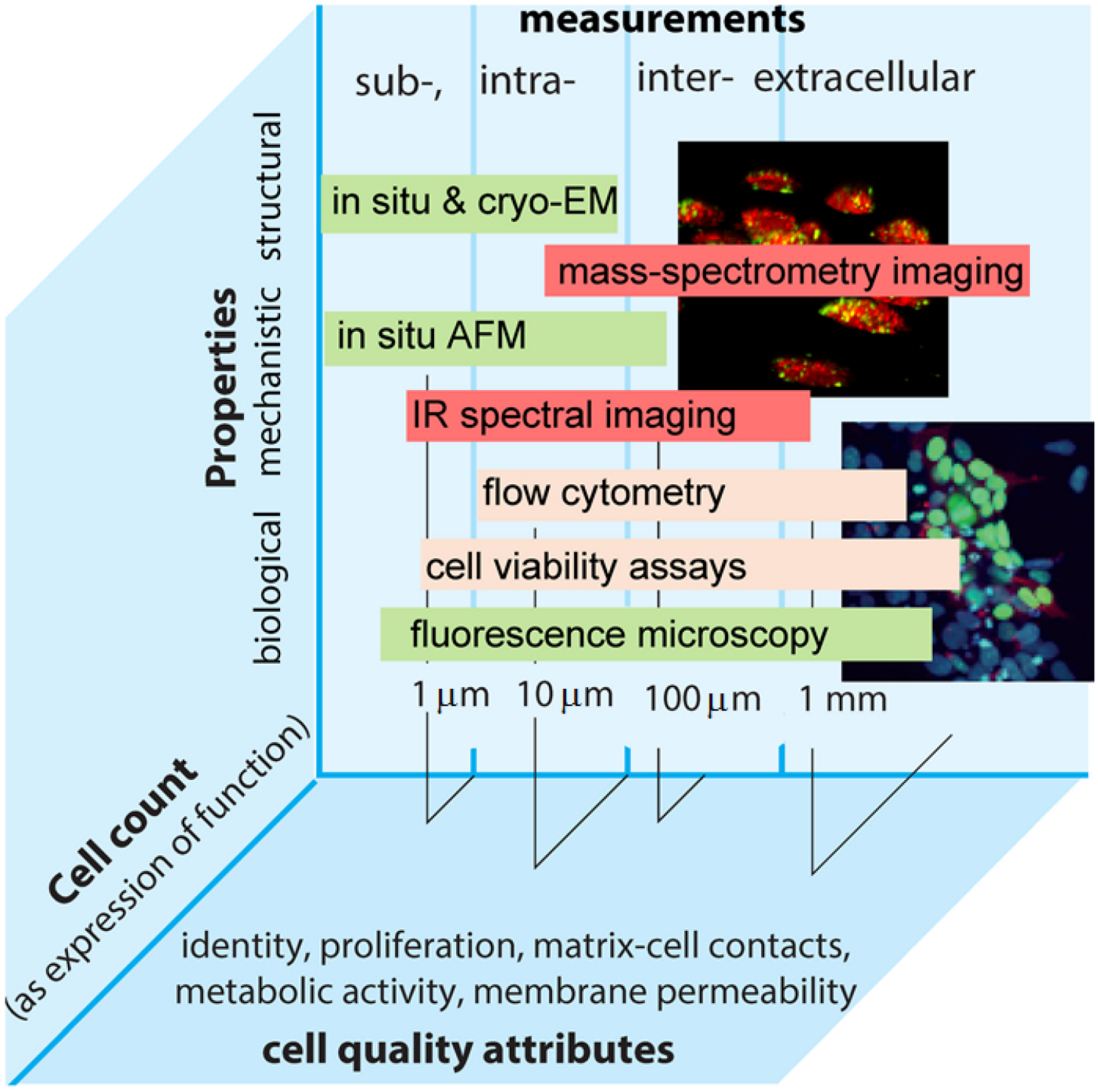
Intra- and extra-cellular measurements performed by different methods in the continuum of biological, structural, and mechanistic properties plotted vs. relevant length scales and cell count as an expression of function. Examples of common cell quality attributes are also given.

Focusing on solving these challenges will help improve reproducibility and traceability in cell measurements. Whilst some areas have advanced with traceability established to the SI—e.g., medical physics and genomics operating at organ and genetic levels—traceable measurements at the cellular level are lacking. These are yet important to address healthcare challenges as well as implement traceability requirements in medicine in light of new regulations (e.g., EU 2017/746). Here key for success is to define appropriate measurands that allow the accurate characterization of cellular behavior and the biological function it underpins (Figure 1).

In principle, cell size, shape, and morphology can be made traceable to the meter. The amount of substance can be expressed as cell count for which the coherent SI unit is the number 1. The expression of function can also be expressed as the number of specific antigens that might be determined, e.g., by counting fluorescence markers (Neukammer et al., 2005). Cell viability provides another important dimension for measurements at cellular levels, though the viability of an individual cell is an ordinal quantity that cannot be traceable to the SI. Nonetheless, this does not exclude traceability in a more generic sense, e.g., by using reference materials or defining viability as a limit of dye permeability.

In this regard, the CAWG coordinates the work of NMIs and Designated Institutes (DIs) aiming at the development of reference materials based on both eukaryotic and prokaryotic cells. Examples include eukaryotic cell reference materials that are designed to support the quantification of blood cells (erythrocytes, leukocytes, thrombocytes) in a blood matrix, e.g., CD4+ (Stebbings et al., 2015) and prokaryotic cell reference materials that target potential impact in the determination of surface material biodegradation for a community of microorganisms, water and food safety.

One consideration for establishing a roster of measurands relevant to the elucidation of structure-function relationships in cell behavior is to identify measurements that elucidate the role of subcellular components (e.g., DNA, proteins, sugars, lipids) critical to manifesting quality attributes of the cell (Lin-Gibson et al., 2016a). Indeed, cells utilize compartmentalization, spatial organization, and dynamic geometric and chemical environments, complex signaling pathways, which all define the measurable attributes of the cell. As such cells express properties of emergent behavior—novel properties that arise from a collection of individual constituents that do not themselves exhibit these properties in isolation—an important consideration when extending cellular analysis beyond purely compositional measurements to functional parameters of intact cells.

Exemplar measurements include identification and quantification of cell number or cell components (e.g., cell surface receptors, *in situ* genes or proteins) and measures of biological response (e.g., cell morphology, gene expression rate). Measurands can thus be conducive to increasing complexity starting with more generic values (e.g., cell count), which is feasible and pursued at the moment (Figure 1) (Lin-Gibson et al., 2016a). Such a bottom-up approach can support metrological traceability with relevance to end-user applications and consequently to measurement services that to date range from a complete blood count and biomarker expression by flow cytometry to cell viability. Metrology institutes already possess capabilities to characterize cell density and confluency fraction of cells and cell shape in given environments, while data on stem cells and dose delivered by specific therapeutic products are on the horizon. Measurements of cell authenticity, viability, and toxicology are already provided as routine contracted services. There are capabilities used to detect rare cells in blood products and characterizing nanoparticles interacting with cells and permeabilizing cells, and capabilities that are technology dependent (e.g., defined by a technology or technique like flow cytometry) as well as technology agnostic measurement services (e.g., quantification of a specific cell type in a given matrix). Involving end-users early in the selection of a particular service proves essential and mutually beneficial for progress toward standardization. For example, cell quantitation remains a paradigm objective of cell metrology. It is necessary to better understand structure-activity, bio-chemical and physical properties of the cell, and establish quantifiable relationships across length and time scales. To enable predictability for product design and ultimately support translational research, such relationships must be free of *a-priori* constraints imposed by the limitations of a particular technique. Therefore, metrology community also coordinates their activities with those of standardization organizations. This helps better align measurement capabilities with real end-user needs, which are also better informed by technology developers. As an exemplar, the launch of novel therapies, including gene and cell therapies, is estimated to reach prescription sales at $1.2 trn in 2024 (Evaluate Pharma, 2018). On the one hand, the emergence of new technologies is rapid, which requires more animal tests and clinical trials. On the other hand, existing and emerging regulatory policies emphasize the lack of suitable standards that limit the use of advanced therapies (EC/1394/2007; EC/2001/83). These factors expose persistent gaps in the availability of higher order reference measurement procedures and reference materials that are necessary to facilitate the translation of innovation into cost-effective products (Eyles et al., 2018). Different organizations have stood up efforts to begin to fill these gaps (e.g., ISO 20391-1:2018, ASTM F2739), while the life sciences community is placing a stronger focus on cell and gene therapies. In terms of functional measurements this emphasis can be broadly grouped into extra- and intracellular measurements.

## Metrology of Extracellular Systems

Different industries are beginning to define the need for specialist measurements and standards for extracellular systems relevant to regenerative medicine, biofilm prevention and microbiome environments. The restoration of damaged tissues and the prevention of infections are among the challenges of the highest priority for healthcare. Indeed, the cost to the National Health Service in the UK for managing a chronic wound alone is conservatively estimated at £5 bn per year (Guest et al., 2016), raising over the last 10 years up to 5% of the total outturn expenditure on healthcare (Posnett and Franks, 2008). In addition, biofilm formation is one of the main contributing factors to chronic wounds (e.g., ulcers) (Sen et al., 2009). Biofilms account for up to 90% of chronic wounds. Although only 6% of these are considered as acute, these are associated with the increasing incidence of diabetes and obesity, which compound the burden of chronic wounds on National healthcare systems (Attinger and Wolcott, 2012; Guest et al., 2016).

The biomedical and life sciences industry develop cell therapeutic products (stem cells), materials that can stimulate tissue restoration (scaffolds, implants) and materials able to prevent or reduce infection and biofilm formation (antimicrobials and their carriers). Barriers to commercialization for innovative technologies include high costs associated with managing tissue treatments (tissue grafts, cell transplants). Measurement needs that derive from these applications include cell assessment as well as interactions of cells with each other through and with their micro-environments or niches provided by scaffolds, culture media and environmental forces (Discher et al., 2009).

One area in the metrology of tissue engineered materials focuses on enabling parameterised interdependencies between cell interactions and forces that cells are exposed to. Combinatory measurements able to monitor cell behavior in response to extracellular environments, both stimulating and detrimental, may provide these interdependencies and improve the understanding of physicochemical properties of extracellular guidance on cell and bioactivity development. These would support controlled cell differentiation in “intelligent” 3D cultures, the assessment of cellular responses in bacteria-challenged environments and would enable regenerative medicine, which unlike conventional and often palliative medicines, offers more comprehensive solutions for tissue restoration. The substantial potential of these medicines in regenerating diseased organs is a major reason for the intensive R&D activities in the field (Rao et al., 2015). The growing markets of tissue treatments are influenced by increasing aging population and patient numbers, while the uptake of promising technologies is bound to more clinical trials (e.g., diabetes, ViaCyte, and spinal cord injury, Geron, USA) thereby impacting on current regulation policies. If regulatory routes can be shortened with relatively small and inexpensive clinical trials new technologies might be commercialized faster. However, shortening regulatory approval times requires justification and reference points against which new technologies can be assessed at early stages, while the availability of reference protocols and materials remains low (Viswanathan et al., 2014).

## Measurement Focus

To this end, inter-laboratory comparisons tend to target cell count as a measurand. Such studies consider microscopic measurements of confluency on two dimensional surfaces in addition to cell count. Both kinds of measurements allow traceability to the International Unit (IU) of 1, but do not reflect the impact of other important cell properties including proliferation, differentiation and viability, which are essential to industries that utilize cells. Developing higher order cell metrology will benefit from pre-defining measured systems and cells with regards to their application relevance (Discher et al., 2009; Viswanathan et al., 2014). In this vein, the community seeks to conduct international comparisons to support global capabilities for technologies such as cell therapies utilizing pluripotent stem cells that can differentiate into a cell or tissue of interest. However, biocompatible substrates are often required to promote the retention of pluripotency and stable replication of stem-cell cultures. Applying a bottom up approach, albeit at this early stage (e.g., cell count), in performing measurements in continuum allows for the provision of the number of stem cells in their proliferative state per unit area (2D) and independently per unit volume (3D). As it stands, measurements could only be traceable to the cell count without capturing the impact of extracellular environments on cell behavior. The importance of the latter is 2-fold. Firstly, tissue development relies on the ability of individual cells to sense and exploit their environments, which in most cases are represented by extracellular matrices (ECMs) (Discher et al., 2009). These matrices support a reciprocal process of tissue development, involving the transduction of biophysical cues to cells and matrix remodeling driven by cells. Therefore, cell-matrix contacts and interactions can provide the most informative measurement targets. Secondly, emerging industry is engaged in developing matrices and scaffolds mimicking native ECMs. Existing mimetics use different chemistries, though an increasing tendency is to mimic the very process of matrix assembly and engineer microenvironments (Rice et al., 2013; Faruqui et al., 2014). Polypeptide self-assembling systems emulate the native ECMs of collagen or fibrin to the point of comparable surface chemistry and are biocompatible and biodegradable. These mimetics give relevant properties of gel formation and topographical cues to nanometer scales but suffer from persistent drawbacks of rigidified nanoscale geometries and narrow size porosities of formed fibrous scaffolds (Rubert Pérez et al., 2015; Lou et al., 2019). As a consequence, they cannot readily respond in dynamic cell culture, but instead confine matrix-cell interactions to local adhesive contacts that fail to support continuous cell recruitment across larger length scales necessary to underpin tissue patterning. The current measurements provide an expression of these contacts in terms of viable cell counts per unit area or volume. This can serve as a starting reference point for the cross-comparison of different cell-supporting matrices, but, naturally, reference matrices that can promote cell adhesion and proliferation in 2D and 3D are necessary to complement this measure (Duval et al., 2017). To help constrain the complexity of biologically relevant studies, metrology research may benefit from efforts by other organizations running international comparisons for non-cell-based higher order reference materials. For example, Versailles Project on Advanced Materials and Standards (VAMAS) supports world trade in products development on advanced materials, while providing the technical basis for harmonized standards and specifications. Similarly, Bureau International des Poids et Mesures (BIPM) maintains the database of higher order reference materials, ranging from drugs to proteins in complex media, which are categorized on the basis of values of the measurands being traceable to the SI (e.g., mass fraction or cell count). In addition, achieving more confidence in measurements is balanced by future considerations that link cell analysis to demands for traceability that accompany emerging technologies. High resolution imaging lacks standard specimens or materials to unambiguously assess instrument performance from the scale of optical imaging to cryo-electron microscopy. For conventional microscopy SI traceable artifacts are available for size measurements in the micrometer range, while for atomic force microscopes nanometre sized traceable calibrators have been proposed (Dixon et al., 2005). The main challenge however remains in providing suitable materials with reproducible morphological or ultrastructural patterns that repeat over multiple length scales. Multi-scale measurements are in the core of cell analysis that is and has to be performed in the continuum of sub-, intra-, inter- and extracellular properties and functions.

## Metrology for Intracellular Systems

Molecular therapies constitute an exemplar domain where quantitative intracellular measurements are urgently needed and may have far reaching benefits (Ryadnov, 2014; Conlon and Mavilio, 2018; Pecot et al., 2018). An ability to deliver gene and macromolecular drugs holds promise for the imminent therapeutic control of major diseases including cardiovascular and genetic disorders as well as cancers. Therapeutics are available. However, their acceptance and application in clinical settings are hampered by uncertainties in intracellular delivery and the structural inconsistency of delivery vectors. Initiatives in industry (e.g., Genzyme Corp.—Erickson's case, Novartis-led global clinical trials of gene treatments for glioblastoma multiforme) build upon the need for more efficient and quantitative intracellular delivery as well as the scalability of vector production (Ginn et al., 2018). Quantitative and correlative measurements of intracellular delivery are anticipated to support the systemic assessment of the safety and efficacy of delivery technologies (Ando et al., 2018). Such measurements rely on parameterised measurands that are relevant to the delivery vectors themselves (Yin et al., 2014). One can adapt the concept of viral titer as a quantitative measure of the vector in a given volume (number of particles per mL). However, viral load testing is performed using gene amplification techniques that are restricted to the total amount of virus often given in RNA or DNA copies per mL. These measurements do not reveal a ratio of functional loaded and empty particles. In comparison, non-viral systems are not as monodisperse as viruses and their size is not strictly tailored to the size of genetic cargo they carry. Although this allows artificial systems to accommodate different genetic cargo, the particles they form are categorized into loaded, over-loaded and empty particles, the ratio of which is also undefined. With no available measurements that can provide an explicit answer with regards to the ratio of loaded vs. empty particles, vector developers accept broad variations of loading. The efficacy of viral and non-viral vectors is linked to their ability to cross cellular membranes and release the cargo without preventing subsequent functions of gene silencing or expression (Yin et al., 2014). Each of these steps has different measurement challenges, whereas an overarching challenge in intracellular delivery is to quantitatively relate the number of gene-delivery particles before and after transfection. Current macromolecular drugs that modulate genetic reactions overcome the problems of stability, excretion and uptake by phagocytes, but the lack of concordant results in gene uptake by the target cells as a function of functional and structural inconsistency of delivery vectors remains unsolved^1^. This is why gene therapy technologies have reached a point where quantitative control over macromolecular transfer is necessary for further progress. To better understand factors improving intracellular delivery, measurements for the quantitative assessment of delivery vectors, their uptake to target cells and specificity of targeting are all important but remain largely untapped by the current development in cell metrology.

## Measurement Focus

An exploitable answer to quantitative gene and drug delivery, at least *in vitro*, can be provided by a reference measure that would incorporate contributions from different measurands. A relatively straightforward way to express it is by combining two key events relevant to any vector candidate—transfection efficacy and genetic reaction (knockdown or expression efficacy of its cargo)—and by normalizing these against the total counts of viable cells at different ratios of cargo to vector (molar or charge). The resulting relative or reference fitness is expressed in arbitrary units, since each event is characterized by a different measurand using a different method, e.g., genetic reactions by PCR against reference genes, transfection efficacy by flow cytometry, microscopy and mass-spectrometry, individually or combined, and cell viability using cell proliferation and viability assays providing quantitative chemical and enzymatic redox indicators of metabolically active cells. All these methodologies have their own limitations that need addressing before they can be combined in continua of correlated measurements (Kim et al., 2007; Pyne et al., 2017). For example, intracellular staining remains an important requirement for cytometry and microscopy, an apparent difficulty for which is to quantify the fixation-permeabilization step, while a similar problem for PCR methods consists in the quantitative extraction of target nucleic acids (He et al., 2016; Cossarizza et al., 2019; Saraiva et al., 2019). Nevertheless, established and emerging techniques and methodologies are already showing encouraging progress in different application areas, providing reference materials and user guidelines, which create a necessary basis for the reference measure of gene delivery. Such a measure can be traceable to a cell count, but as in the case of extracellular measurements does require supporting reference materials, which in this case are gene delivery vectors. The criteria for a meaningful candidate are also relatively well understood. One has to ensure that the reference fitness is reproducible, i.e., the candidate has to be able to transfect cells without cytotoxicity in a wide range of concentrations and ratios with its cargo and has no affinity to the cargo following cytoplasmic release (Yin et al., 2014). Additional requirements are structural monodispersity, avoiding aggregation or agglomeration effects, neutral or close to neutral surface charge, and accessible surface chemistry for potential modifications as determined by requirements for specialist reference materials on its basis. In conjunction with that viruses remain the most effective transfection reagents, these criteria are best met by a biopolymer shell (polypeptide or polysaccharide) that is assembled by following the principles of the virus architecture. Research in this area, both in academia and industry, is significant and provides an improved set of structure-activity principles that can guide the engineering of a reference material to support the advancement of gene therapy (High and Roncarolo, 2019). There is also a number of suitable candidates under consideration by some metrology institutes, which are ripe for inter-laboratory comparability studies (De Santis et al., 2017; Zhao et al., 2017). What remains to be achieved is a consensus concerning the reference measure, what it incorporates and what measurement capabilities are most appropriate to characterize a proposed material. The choice of an appropriate cell line is a fundamental variability in cell analysis (Freedman, 2015; Lin-Gibson et al., 2016a). This is unlikely to be solved in any foreseeable future, but can be pre-defined and endorsed by stakeholder communities whose products are typically developed against specific cell lines. Reference materials are indispensable components for establishing traceability and such a joint approach will accelerate the establishment of a reference system that can deployable to the needs of gene therapy and manufacturing.

## Future Outlook: Focusing On End-User—Translational Cell Metrology

The mission of establishing a strong infrastructure supporting reproducibility and traceability in cell measurements is driven by impact for the end user. A lack of measurement assurance infrastructure—including traceability to the IU—is associated with the issues of reproducibility which are of immediate importance to cell metrology. The state of irreproducible results in biological measurements and their impact on industry is well documented. For example, in 2012 Amgen reported that only six out of 53 preclinical studies could be repeated (Begley and Ellis, 2012). In the subsequent year, Global Biological Standards Institute (GBSI) released the “Case for Standards” (Global Biological Standards Institute, 2013). This case is one of the first comprehensive reports addressing irreproducibility issues in biomedical research and underpins the Reproducibilty2020 action plan that outlines priorities in the development of standards for biological reagents, cell culture, sera validation, cell assays as well as laboratory protocols, many of which are underpinned with the fundamentals of cell metrology (Freedman et al., 2017).

Creating an international metrology network providing a sustainable access to measurement and standards that support regulation and the availability of measurement services may accelerate all these developments and their implementation for the end-user needs. To this effect, working groups in CCQM provide vehicles to advance measurement assurance principles and establish a means to metrological traceability. Given that cell metrology is at early days to be able to fully address the complexity of cellular measurements, there is an increasing tendency to place a stronger emphasis on property related efforts thereby setting priorities on particular cellular processes or cell-based products. This will also influence a reciprocal end-user view in addressing fundamental questions that evolve around the lack of and need for traceability in the life sciences sector (Badrick et al., 2018). Coupled with regulations that establish more stringent criteria for traceability through the supply chain and are driven by requirements to better protect public health and patient safety, the metrology of cell-based systems is well-positioned for positive impact (Calvo et al., 2018). Concomitantly, consensus standards strive to provide clarity for regulatory expectations for pre-market submissions via a declaration of conformity or for general use (e.g., FDA guidance on voluntary consensus standards). By focusing on reference measurement systems, the CAWG brings up the need for comparability of measurement results in a continuum of pertinent properties and is striving to understand the value of reference materials and other measurement services for method harmonization where appropriate. This can also mitigate the growing risks of the replication drive that threatens to compromise promising research results due to the lack of reference systems that can fully address the complexity of cellular measurements (Bissell, 2013; Freedman, 2017).

All in all, a stronger response to end-user needs at an early stage of cell metrology developments through focused efforts of individual metrology institutes and engaging in consensus standards activities, while taking into account the complexity of cellular measurements can provide a significant step change in cell measurements with far-reaching benefits to stakeholders. In particular, this is the case for small and medium size enterprises that operate at early, high-risk stages, where there exist severe gaps in measurement infrastructure support making it difficult for them to survive. On a more fundamental scale where cell metrology may demonstrate its share of value, this strategy will allow metrology institutes to impact on reverting the current prevalence of irreproducible preclinical research, which is estimated to exceed 50%, with associated annual costs being in the range of $28 bn in the United States alone (Freedman et al., 2015).

## Author Contributions

All authors listed have made a substantial, direct and intellectual contribution to the work, and approved it for publication.

### Conflict of Interest

The authors declare that the research was conducted in the absence of any commercial or financial relationships that could be construed as a potential conflict of interest.

## Footnotes

^1^FDA Guidance for Industry. *Gene Therapy Clinical Trials; EMEA Guidance on the Quality, Preclinical and Clinical Aspects of Gene Transfer Medicinal Products*. EMEA/273974/05.
